# Cancer-associated fibroblasts as abettors of tumor progression at the crossroads of EMT and therapy resistance

**DOI:** 10.1186/s12943-019-0994-2

**Published:** 2019-03-30

**Authors:** Micol Eleonora Fiori, Simone Di Franco, Lidia Villanova, Paola Bianca, Giorgio Stassi, Ruggero De Maria

**Affiliations:** 1 Department of Oncology and Molecular Medicine, Istituto Superiore di Sanità, 00161 Rome, Italy; 2 Department of Surgical Oncological and Stomatological Sciences, University of Palermo, 90127 Palermo, Italy; 3 Istituto di Patologia Generale, Università Cattolica del Sacro Cuore, Largo Francesco Vito 1, 00168 Rome, Italy; 4 Scientific Vice-Direction - Fondazione Policlinico Universitario “A. Gemelli” - I.R.C.C.S, Largo Francesco Vito 1-8, 00168 Rome, Italy

## Abstract

In the last decades, the role of the microenvironment in tumor progression and therapeutic outcome has gained increasing attention. Cancer-associated fibroblasts (CAFs) have emerged as key players among stromal cells, owing to their abundance in most solid tumors and their diverse tumor-restraining/promoting roles. The interplay between tumor cells and neighboring CAFs takes place by both paracrine signals (cytokines, exosomes and metabolites) or by the multifaceted functions of the surrounding extracellular matrix. Here, we dissect the most recent identified mechanisms underlying CAF-mediated control of tumor progression and therapy resistance, which include induction of the epithelial-to-mesenchymal transition (EMT), activation of survival pathways or stemness-related programs and metabolic reprogramming in tumor cells. Importantly, the recently unveiled heterogeneity in CAFs claims tailored therapeutic efforts aimed at eradicating the specific subset facilitating tumor progression, therapy resistance and relapse. However, despite the large amount of pre-clinical data, much effort is still needed to translate CAF-directed anti-cancer strategies from the bench to the clinic.

## Introduction

Solid tumors can be considered as aberrant organs, which have undergone molecular and cellular reprogramming, promoting a proliferative and invasive niche, ideal for cancer cell propagation and homing at metastatic sites. Like healthy organs, tumors are characterized by high cellular heterogeneity, not only within the transformed cell compartment (i.e. cancer stem cells, progenitor and differentiated cancer cells). Indeed, tumors contain peculiar cellular and non-cellular components, which altogether form the tumor microenvironment (TME). This complexity is a major hurdle in the understanding of the mechanisms responsible for treatment failure. Cell types within the TME include: neuro-endocrine, adipose, endothelial, mesenchymal, immune-inflammatory cells as well as fibroblasts [1]. Among stromal cells, fibroblasts are particularly important because of their abundance (up to 80% of the tumor mass in pancreatic tumors [2]) and their robust crosstalk with cancer cells. Fibroblasts, which are usually quiescent, can be reversibly or irreversibly activated in response to different inputs occurring upon tissue damages, generating the normal activated fibroblasts (NAFs), also called myofibroblasts and characterized by the expression of α-smooth muscle actin (α-SMA), a marker of smooth muscle cells [3]. Recent data show that during the acute inflammation process the reversible activation of NAFs is mediated by the presence of growth factors. In contrast, in chronic inflammation the acquisition of epigenetic alterations locks NAFs in a state of irreversible activation [4]. When fibroblasts’ activation persists even in absence of the initial insults, they can promote tumor initiation. It has been widely demonstrated that cancer cells interact with fibroblasts during all stages of disease progression. Fibroblasts associated with cancer have been named CAFs (reviewed in [4, 5]).

CAFs can derive from different cell types, such as NAFs, epithelial cells following EMT, endothelial cells via endothelial-to-mesenchymal transition (EndMT), bone marrow-derived cells (BMDCs), adipocytes and stellate cells [6]. They are characterized by increased expression of markers such as α-SMA, fibroblast activation protein (FAP), fibroblast specific protein 1 (FSP1 or S100A4), VIMENTIN, and platelet-derived growth factor receptor (PDGFR)-α and β [5]. Unfortunately, none of these markers is specific to this cell subpopulation, which is characterized by a high grade of heterogeneity, thus making it more difficult to study CAFs’ role in different pathological contexts.

### Heterogeneity of Cancer-associated fibroblasts

Tumor heterogeneity, which is considered the driver of current anti-tumor therapies’ failure, involves both the transformed epithelial cells and the stromal cellular components. This heterogeneity originates from intrinsic (i.e. different cellular phenotype) and extrinsic factors (i.e. tumor progression, treatments and spatial distribution). Consequently, tumor cells are exposed to different signals in primary tumor versus metastatic environment, in small versus large lesions, in the center versus the invasive front. These findings have an important clinical value, as cancer cells may be confined, in different steps of tumor progression, in a favorable or hostile environment that shapes their behavior and therapeutic response. Therefore, elucidating the mechanisms underlying this stromal heterogeneity may have a strong impact on the prognosis of cancer patients and lay the foundations for the development of new therapeutic protocols.

In this scenario, Ohlund and colleagues have reported in pancreatic ductal adenocarcinoma (PDAC) the existence of distinct subsets of CAFs with different localization within the tumor. In particular, the authors identify α-SMA^high^ CAFs in direct contact with neoplastic cells, while α-SMA^low^ CAFs localize distant from cancer cells and display a strong paracrine release of pro-inflammatory cytokines, including IL-6 [7].

A recent study by Costa and colleagues demonstrates the existence of four CAF subsets (S1–4), with unique properties and activation levels, which accumulate differently in breast cancer subtypes (Luminal A, HER2 and Triple negative) [8]. In particular, by using six CAF markers (CD29, FSP1, FAP, αSMA, PDGFRβ and Caveolin1), the authors show that S1-CAFs are associated with an immunosuppressive tumor microenvironment by attracting T cells and promoting their differentiation into T-reg, in contrast to S4-CAFs that are associated with high CD8^+^ T cell infiltration. Further, Neuzillet and colleagues have confirmed by transcriptomic analysis in PDAC the classification of CAFs into four subsets (subtypes A-D) found in breast cancer [9]. As previously demonstrated, each subtype possesses a specific phenotype and a prognostic impact. All four subsets express ECM-related genes, while immune-related pathways are selectively enriched in subtype C. Importantly, this classification correlates with the one found in lung cancer by Lambrecht et al., supporting the concept of fibroblasts’ intra-tumor heterogeneity with in vivo spatially distinct CAF subsets within single tumors [10]. The authors have identified specific markers to label three out of the four CAF subsets, with Periostin as a marker of subtype A (found at the invasive front of primary tumor and crucial to the formation of tumor capsule and metastatic niche), Myosin-11 for subtype B (enriched in larger tumors characterized by lymph node metastases and poor prognosis), and podoplanin in subtype C (immunogenic tumors).

In another study, Su et al. identify a specific subset of CAFs, characterized by the expression of CD10 and GPR77 and persistent NF-kB pathway activation, which promotes tumor formation and chemoresistance in breast and lung cancer [11]. In oral squamous cell carcinoma (OSCC), Costea and colleagues put in evidence the presence of two CAF subsets, with the CAF-N population characterized by a phenotype and paracrine activity more similar to normal fibroblasts, and the CAF-D counterpart showing a different expression pattern and high release of TGF-β [12]. Noteworthy, the inhibition of CAF-N, intrinsically more motile, impairs the invasion of adjacent OSCC cells, while neutralization of CAF-D function by TGF-β blockade impairs keratinocytes’ EMT and invasive potential. This study postulates the occurrence of two CAF subtypes both promoting OSCC invasion by acting on different molecular mechanisms of cancer cells.

Even in absence of a molecular or phenotypic characterization, the existence of CAFs restraining tumor growth has been first hypothesized in pancreatic cancer. Two back-to-back reports have jointly demonstrated that erasing α-SMA-expressing myofibroblasts in two different genetically engineered mouse models (GEMM) of PDAC resulted in a more aggressive tumor and did not improve gemcitabine’s efficacy, owing to suppressed immune surveillance and increased tumor vascularization, respectively [13, 14]. More in detail, Ozdemir and colleagues show that overall ablation of α-SMA^+^ fibroblasts leads to more invasive and undifferentiated tumors, more pronounced hypoxia, and concomitant induction of EMT and cancer stem cells (CSCs) enrichment. Importantly, the authors also notice an enrichment in FoxP3^+^ T-reg cells upon CAFs depletion and administration of an anti-CLTA4 antibody significantly improved mice survival [13]. Similarly, Rhim et al. demonstrate that Shh-deficient PDAC mice harbor more aggressive and undifferentiated tumors with a reduced number of α-SMA^+^ myofibroblasts and increased vascularization [14]. Moreover, a recent work by Patel et al. identifies in oral carcinoma two CAF subsets characterized by different levels of α-SMA expression, the α-SMA^-^ (C1) and α-SMA^+^ (C2) [15]. In particular, C1 CAFs positively regulate proliferation and concomitantly suppress self-renewal of oral cancer cells by releasing BMP4, as compared to the C2 subset. In line with these data, Brechbuhl et al. describe two CAF populations that differentially express CD146 and play conflicting roles in affecting efficacy of endocrine therapy in luminal breast cancer [16].

Taken together, these findings suggest that a better characterization of CAF subtypes and their specific role in tumor progression could offer innovative therapeutic tools for the development of anti-tumor treatments. Notwithstanding, these results also entail the need for caution in targeting CAFs in cancer patients, suggesting that a combinatorial rather than a single-agent therapy could be more effective.

Despite the very recent evidence regarding the presence of CAFs endowed with anti-tumorigenic potential, CAFs are well known for their role in the establishment of favorable conditions for in situ tumor growth and metastatic spread of cancer cells [17]. Among the plethora of mechanisms regulated by CAFs in tumor progression, the modulation of cancer stemness, EMT and therapy resistance has direct repercussions on oncologic patients’ survival. In this scenario, we will review here the most recent findings regarding CAFs-mediated metastatic behavior and resistance to therapy.

## Mechanisms of CAF-mediated control of tumor progression

Within the tumor bulk, the more undifferentiated cancer cells can fluctuate between different states due to their plasticity, which has been reported as a peculiarity of CSCs, together with tumorigenic potential and self-renewal [18]. Initially, CSCs were isolated and characterized in acute leukemia [19, 20] and then they were identified in many other cancers [21]. The interest of the scientific community in this cellular population originates from growing evidence that supports its involvement in crucial steps of tumor progression, including tumor initiation and growth, metastases formation, drug resistance and relapse, being responsible for minimal residual disease (MRD). Cancer stemness and mesenchymal phenotype have recently been demonstrated to strongly correlate. Indeed, it has been observed that cancer cells that acquire EMT traits gain CSC-like properties, and CSCs often undergo EMT in order to generate metastases [22–24]. In fact, the EMT process can be crucial during the dissemination step that precedes metastatic colonization [25]. However, the transition between an epithelial- to a mesenchymal-like phenotype is not a sharp switch, but rather occurs through different steps, thus defining a gradient of metastable phenotypes, where specific mesenchymal and epithelial features coexist and eventually lead to the acquisition of a stable EMT programme [26]. During the first stage, characterized by a continuous source of stimuli driving the acquisition of the mesenchymal state, we observe the activation of specific pathways driving the EMT, which can be reverted once the TME stimuli cease. Differently, gaining a stable EMT phenotype includes a gene expression reprogramming, which involves the activity of specific transcription factors, non-coding RNAs or epigenetic changes, and it often occurs as a result of prolonged exposure to stimuli driving EMT [27]. It is clear that CAFs can regulate EMT in cancer cells, however the underlying mechanisms are not completely understood. Here, we summarize the most recent findings regarding the crosstalk that defines the cooperation between CAFs and cancer cells in different phases of tumor progression. Such interplay can occur through different mechanisms, including CAFs’ altered secretome, which consists of growth factors and cytokines directly involved in the positive regulation of cancer cell survival, proliferation, stemness, and resistance to therapy. Moreover, by releasing cytokines and matrix metalloproteinases (MMPs), CAFs enhance tumor angiogenesis, local inflammation and extra-cellular matrix (ECM) stiffness.

### CAFs paracrine effects

One of the most studied CAFs-released cytokines is the transforming growth factor-β (TGFβ), whose pathway is crucial in driving tumor progression in different cancer models [28]. TGFβ binds a complex of transmembrane receptor serine/threonine kinases (types I and II) and induces trans-phosphorylation of the type I receptor by the type II receptor kinases. Activated type I receptors phosphorylate Smad2/3 and these receptor-activated Smads (R-Smads) form a complex with the common-mediator Smad (co-Smad) Smad4. Activated Smad complexes translocate into the nucleus, where they regulate transcription of target genes by cooperating with DNA-binding transcription factors and coactivators (canonical signaling) [29]. In addition, TGFβ is also able to regulate other cancer-related pathways, including MAPK and PI3K/Akt, through the non-canonical signaling [30]. TGFβ-driven effects have been demonstrated to be highly cell-type dependent [31]. Although it exerts a dual role during different phases of tumor progression, TGFβ pathway gained a great consideration in oncology since it has been found deregulated in many cancers [32]. In healthy tissues and in early stages of tumor formation, TGFβ activation plays a protective role inducing cell-cycle arrest and apoptosis [33], whereas in advanced cancer it regulates the acquisition of a mesenchymal phenotype, hence being a driver of the metastatic disease [34]. In addition to its involvement in the regulation of EMT [35, 36], it has been reported a direct link between activation of TGFβ and cancer stemness [37, 38]. Zhuang et al. have recently shown that TGFβ1 is highly present in CAF-conditioned medium (CAF-CM) and induces EMT in bladder cancer cells by activating the canonical TGFβ signaling through the activation of Smad2 [39]. In this model, TGFβ is sufficient to induce over-expression of EMT-related genes, including *VIMENTIN*, *FIBRONECTIN*, *SNAI1*, *ZEB1* and *ZEB2*. The authors have demonstrated that this cancer cell reprogramming is driven by the up-regulation of a long non-coding RNA (lncRNA), ZEB2NAT, a natural antisense transcript of *ZEB2*. In line with these findings, TGFβ pathway has been shown to control the epigenetic signature of cancer cells by up-regulating the lncRNA HOX transcript antisense RNA (HOTAIR) in breast cancer [40]. HOTAIR mediates H3K27 tri-methylation with consequent silencing of tumor suppressors in many cancer types [41], including breast cancer, where it is reported to promote drug resistance and cancer stemness [42]. Here, Ren and colleagues demonstrate that the TGFβ1/HOTAIR axis, by targeting CDK5 signaling, promotes the metastatic capacity of breast cancer cells, thus suggesting that its targeting may be considered a novel strategy for the treatment of breast cancer. The pronounced secretion of TGFβ1 by CAFs in breast cancer promotes an aggressive phenotype in tumor cells also through direct activation of EMT, with decreased expression of E-CADHERIN and over-expression of VIMENTIN, Fibronectin1 (FN1), MMP2 and MMP9 [43]. Enhanced TGFβ signaling has been identified in CAFs from colorectal cancer subtypes with poor prognosis, as part of a stromal signature that correlates with disease relapse. TGFβ-activated fibroblasts actually promote tumor initiation in functional assays and administration of a TGFβR1-specific inhibitor in a metastatic mouse model of colorectal cancer impairs the capacity of tumor cells to thrive in the liver over the colonization phase [44].

Other important signaling pathways that drive the gaining of mesenchymal traits are MAPK, PI3K/Akt, Wnt/β-catenin and JAK/STAT [45]. These pathways are regulated by growth factors and inflammation mediators commonly released by CAFs, including hepatocyte growth factor (HGF) [46], stromal-derived factor-1α (SDF1) [47], osteopontin (OPN) [48], fibroblast growth factor (FGF) [49], interleukin-6 (IL-6) [50]. We have identified HGF, SDF1 and OPN as the key cytokines released by CAFs able to reprogram colorectal cancer cells toward CSCs endowed with metastatic potential. Briefly, such CAF-derived signals induce expression of the functional CSC marker CD44v6 through activation of the Wnt/β-catenin signaling pathway, which fosters migration and metastasis [24]. Lineage tracing of colorectal CSCs in mouse xenografts performed by Lenos KJ et al. has further highlighted the role of CAFs in conveying stem cell functionality (meant as clonogenic capacity) to neighboring cells at the invasive edge of the tumor. Notably, the most abundant secreted factor expressed in murine CAFs was OPN, and xenografts derived from OPN-overexpressing CSCs displayed a homogeneous distribution of clonogenic cells throughout the tumor bulk, with no significant difference between centre and edge. An important implication of this study is that non clonogenic cancer cells can acquire self-renewal ability as soon as they gain access to the right niche, enriched in CAF-secreted OPN [48].

CAF-derived HGF promotes cancer cell tumorigenic and metastatic potential by activating the HGF/c-MET pathway [51]. In this work, Ding and colleagues unveil the effects of CAFs-released HGF in the promotion of proliferation, migration and invasion in *MET*-unamplified gastric cancer cells. HGF ligand, by binding the c-MET receptor, drives a plethora of intracellular signaling pathways that regulate several aspects of tumor cells, including survival, stemness, EMT, dissemination and clonogenic potential [52]. The versatile biological effect of HGF in cancer cells is given by its interaction and cooperation with other crucial pathways (MAPK, PI3K/Akt, JAK/STAT) that are considered as drivers of tumor initiation and progression. In fact, by regulating the expression of IL-6R, HGF also activates the IL-6/IL-6R/JAK2/STAT3 pathway that in turn augments the expression of c-MET with a positive feedback regulation [51]. The coordination of these two pathways drives tumorigenic progression of cancer cells in response to CAFs’ paracrine activity. IL-6 is an inflammatory cytokine that binds its membrane receptor IL-6Rβ (gp130) that, upon dimerization with IL-6Rα, activates the intracellular JAK/STAT pathway. As for other cytokines, IL-6 driven-effects are also extended to other pathways, thus regulating several biological responses in target cells, including the activation of MAPK, PI3K, and Notch, which play an important role in inflammatory disease and cancer development [53]. In addition, IL-6 serves as a platform to recruit immune cells to tumors and enhance the production of pro-inflammatory cytokines, promoting a chronic inflammatory environment.

Further, the regulation of PI3K/Akt in cancer cells by CAFs has recently been investigated by Yu et al., who demonstrate that the secretion of periostin (POSTN), by binding the Protein tyrosine kinase 7 (PTK7), increases cell proliferation and invasion of head and neck cancer cells [54]. PI3K activation arises from the binding of growth factors or cytokines to the cell surface receptor tyrosine kinase (RTK). This leads to the intracellular activation of the catalytic subunit p100 that in turn forms heterodimers with the regulatory subunit p85, triggering the formation of phosphatidylinositol-3,4,5-trisphosphate, PI(3,4,5) P3 (PIP_3_), a second messenger that activates several downstream signaling molecules, including AKT. Once activated, AKT can phosphorylate and activate its downstream effectors including GSK3, FOXO or mTOR regulators. This pathway controls several aspects of cancer cells behavior, including proliferation, metabolism, EMT and survival [55].

All these clinical and preclinical studies demonstrate the need to target the interaction between cancer cell surface receptors and stromal-secreted factors in order to ameliorate the outcome of cancer patients.

### Cancer cells-mediated CAFs reprogramming

**Fig. 1:**
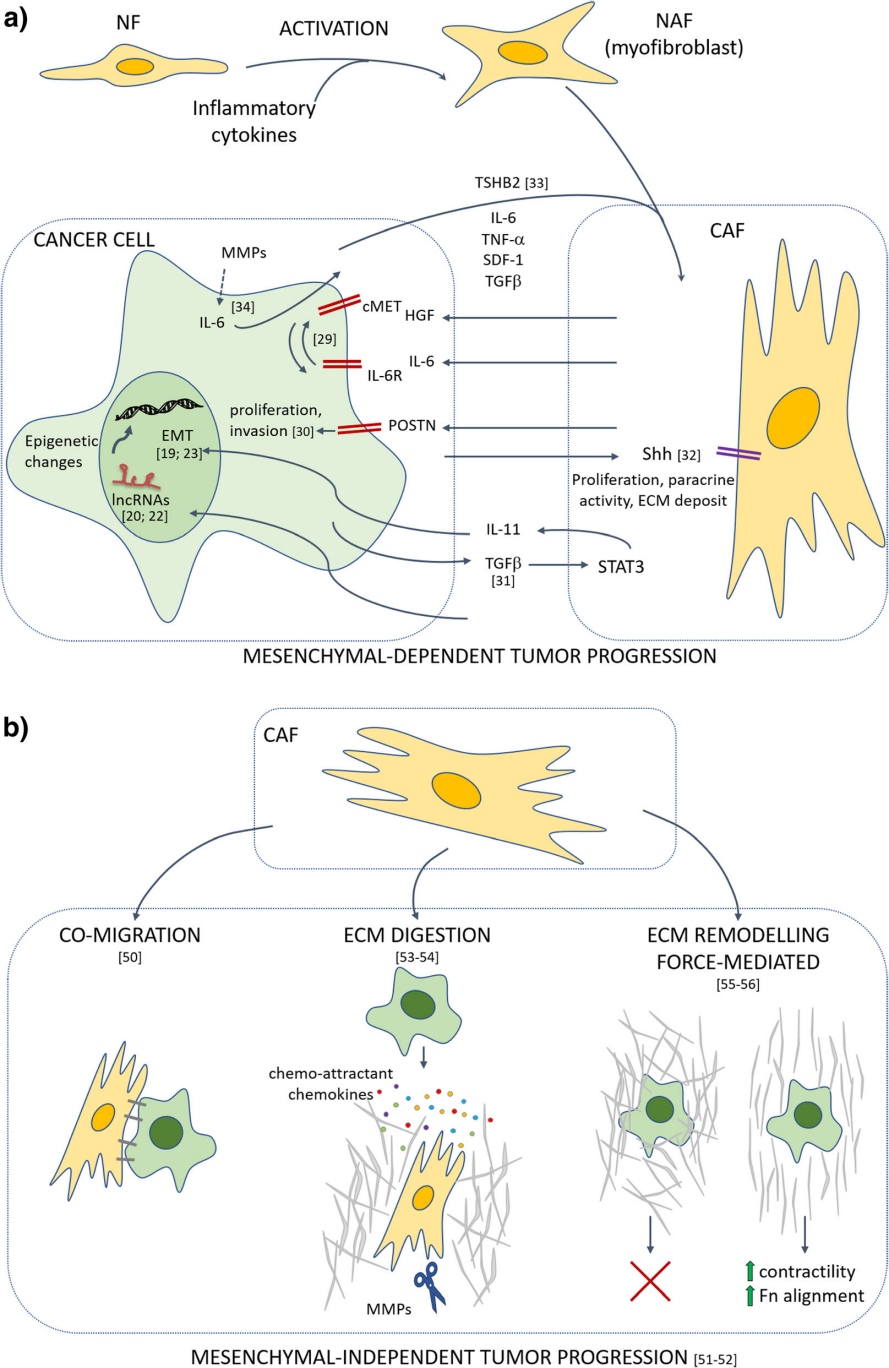
Schematic diagram showing the effects of CAFs on cancer cell metastatic behavior. **a**) Activated fibroblasts (NAF) originate from normal fibroblasts (NF) upon exposure to inflammatory cytokines. Following contact with cancer cells, they can originate the cancer-associated fibroblasts (CAFs) with enhanced proliferative and paracrine potential. The paracrine activity of CAFs and cancer cells underlying the bidirectional crosstalk between the two cell populations with the specific involved deregulated pathways are depicted. The arrows indicate the stimulatory effect of each cytokine. The induction of EMT in cancer cells relies on the activation of transcription factors, lncRNAs and epigenetic changes. **b**) CAFs-mediated effect on mesenchymal-independent (cancer cells maintain an epithelial-like phenotype) invasive potential. Different strategies are adopted by CAFs to facilitate cancer cells invasion of ECM, thus favoring their metastatic potential. Among these, we find the co-migration, by which CAFs and cancer cells migrate together thanks to the expression of cell membrane junctions; the ECM digestion that consists in the production of proteases by CAFs that is accompanied by the release of chemokines acting as chemoattractants for cancer cells; the force-mediated ECM remodeling that consists in the augmented contractility of the ECM and the concomitant alignment of Fibronectin (Fn), thus offering to the cancer cells a preferential route in the invasive process

The crosstalk between CAFs and cancer cells, in particular CSCs, has been described as bi-directional. CSCs not only drive the transition of normal fibroblasts (NFs) into CAFs, but they also hijack fibroblast activity for their own benefit. Beside its key role in driving the EMT in cancer cells, TGFβ has been shown to reprogram also CAFs. Calon and colleagues have demonstrated for the first time that TGFβ released by colon cancer cells activates STAT3 pathway in stromal cells, which in turn enhance their secretion of IL-11 that increase the metastatic potential of cancer cells [56]. In a recent work, Valenti et al. demonstrate that CSCs, which are preferentially located at the tumor-stroma edge, secrete Sonic Hedgehog (SHH) that in turn stimulates the Hedgehog signaling in adjacent CAFs, thus leading to a boost in their proliferative potential, growth factors release (IGF-1, ACTIVIN A, NOV and LIF) and ECM deposition [57]. Although the presence of CAFs and their interplay with cancer cells has been observed in both primary tumor growth and distant metastases, their role in different steps of tumor progression is still object of investigation. Accomplishment of the metastatic colonization process requires the EMT phenotype to be switched off once cancer cells are seeded in distant sites, in order to give rise to macrometastases. Metastasis initiating cells (MICs), which originate from the primary tumor, are characterized by a partial and reversible mesenchymal-like phenotype and have been reported to strongly activate metastasis-resident fibroblasts [58]. Del-Pozo-Martin and colleagues have recently demonstrated that in the first phase of metastatic niche induction in breast cancer, AXL^+^ MICs activate fibroblasts by thrombospondin 2 (THBS2) release. This is followed by enhanced BMP signaling activation and TGFβ down-regulation that promote the acquisition of an epithelial-like phenotype, necessary for the metastatic establishment in the second phase of the process [58]. A further demonstration of the importance of the mutual interplay between cancer cells and CAFs has been provided by Giannoni et al., who have demonstrated that MMPs released by CAFs induce the expression of IL-6 in mesenchymal prostate cancer cells, which in turn activates CAFs [59] (Fig. 1a).

### CAFs role in ECM remodeling

The extracellular matrix is a complex network of macromolecules such as collagens, elastin, fibrin and proteoglicans. ECM confers support to the tissues and aids in maintaining their architecture and integrity, contributing to their functional roles with a tight regulation of cell growth, migration, protein synthesis and secretion [60]. ECM structure undergoes constant remodeling, maintaining the balance between synthesis and degradation. ECM functions as a barrier, anchorage site, movement track, but it can also initiate or regulate signaling events by interacting with various cell surface molecules including integrins, syndecans and discoidin domain proteins [61]. Integrins and their associated RTK are involved in cellular response to biochemical and physical changes. In ECM there are also non-structural proteins, which act as precursors of signaling molecules and proteins called matricellular proteins [62] capable of modulating biological processes in a context-dependent fashion, including thrombospondin1–2 (TSP), secreted protein acidic and rich in cysteine (SPARC), tenascin C, and osteopontin [63].

ECM is a dynamic system that under pathological conditions alters its physical and biochemical properties, such as its elasticity and molecular composition [64]. Altered ECM is a common condition in cancer and it has been shown to be required for cancer progression [65]. Stromal cells in proximity of the ECM, including CAFs, immune cells and mesenchymal stem cells, orchestrate a sophisticated program based on cell-ECM interactions in both physiological and pathological conditions [47, 66]. These cells contribute to ECM remodeling by secreting important proteases such as MMPs [67]. In physiological conditions, the main role of fibroblasts is to produce components of ECM as fibronectin, type I, III, V collagens, which are indispensable components of connective tissue, maintaining ECM homeostasis and turnover. Besides the demonstrated up-regulation of type I, III, V collagens, proteoglycans and glycosaminoglycans, the transition of NFs to CAFs promotes the deposit of collagens IV, VII, XI, and XV [68]. The increased deposition of collagens contributes to the ECM stiffening. This process matches with higher activity of LOX-like proteins that are responsible of establishing both intra- and inter-molecular covalent crosslinking of collagen, by oxidative deamination of specific lysine and hydroxylysine residues [65, 69]. The remodeling of the extracellular matrix, represents one of the most important features of cancer progression. Indeed, numerous MMPs are shown to be over-expressed in different types of tumors. MMP3 over-expression in CAFs was observed in mammary glands [70], MMP2 is over-expressed in gliomas [71], whereas MMP1 was observed to increase in melanoma [72] and breast cancer [73]. Moreover, CAFs are actively involved in the secretion of proteases, like uPA, which can cleave and activate MMPs [74].

By cell-cell contact, CAFs are also reported to exert a physical momentum that regulates cancer cell invasion, as highlighted by the observed collective invasion and migration of CAFs and cancer cells [75]. Carcinomas can retain an epithelial phenotype during tumor progression that limits the degradation and invasion of the ECM [76, 77]. In this context, the possibility to develop a cooperative invasive strategy could be decisive for the success of the metastatic process. This partnership in crime of CAFs and cancer cells for the formation of distant metastatic foci takes place through different strategies. CAFs can remodel the ECM thus creating the path for cancer cells to migrate [78]. Moreover, cancer cells can simply follow CAFs during migration through the ECM, being in steady communication due to the secretion of chemokines that generate a chemotactic gradient. This process has recently been investigated by Neri et al., who demonstrated that mesenchymal-like cancer cells increase the matrix-remodeling ability of CAFs, thus leading to the joint invasion of both CAFs and cancer cells [79].

Although physical ECM remodeling is crucial to allow cancer cells’ migration, it has been proposed a different and more complex mechanism in which ECM remodeling is force-mediated. For instance, the numerous attachment-points allow CAFs to transmit a mechanical force to ECM, driven by Myo II-contractility [80]. A recent study by Erdogan and collaborators shows that Fibronectin, which is highly expressed by CAFs, promotes migration of cancer cells [81]. Briefly, CAFs are implicated in ECM remodeling by promoting the alignment of high amounts of Fibronectin in parallel fibers, which guide the cancer cells in their directional migration. In particular, the over-expression of Myosin II and PDGFRα by CAFs, through the α5β1 integrin, leads to an augmented contractility and traction force. In this process, α5β1 acts as a mechanotransducer, while PDGFRα enhances its activity (Fig. 1b).

## Mechanisms of CAF-mediated therapy resistance

As already discussed, the complexity of cancer does not rely merely on intrinsic features of tumor cells. Rather, the interconnections between transformed cells and different components of the tumor microenvironment exert a pivotal role in cancer onset, homeostasis, spread and response to insults such as nutrient/oxygen deprivation or therapeutic drugs. Recent studies have reported an increase of the stromal compartment in colorectal and breast cancer of chemo-treated patients [11, 82]. This phenomenon has been recapitulated in mouse models where resistant tumor xenografts display a larger stromal compartment [83]. These observations imply a putative role of the TME in promoting the adaptive response to therapeutic pressure. Indeed, chemotherapy-induced activation of the stromal compartment supports the survival of residual cancer cells by fostering pro-survival pathways, stemness traits and/or metabolic reprogramming and partially accounts for tumor resistance and recurrence [84]. Specifically, sustained NF-kB activation in CAFs exerts a crucial role in orchestrating the molecular mechanisms underlying their tumor-supportive function upon therapeutic insults, through the release of paracrine signals such as cytokines, exosomes and metabolites [11, 84–86]. Unraveling the crosstalk of cancer cells with TME is therefore compulsive in order to identify novel therapeutic approaches and to overcome resistance to the existing regimens. Notably, non-transformed components of the tumor are genomically more stable than transformed cells, entailing a more durable response to drugs and candidating tumor stroma as an appealing therapeutic target.

### Secretion of cytokines

Under therapeutic pressure, cytokines released by CAFs mediate the activation of different signaling cascades in tumor cells leading to resistance and eventually relapse.

In prostate cancer, DNA damage induced in CAFs upon exposure to chemotherapy triggers transcriptional activation of WNT16B via NF-kB [85]. WNT16B acts as a paracrine signal that activates the canonical Wnt program in tumor cells, which mitigates the effects of cytotoxic chemotherapy in vivo in favor of disease progression. In pancreatic ductal adenocarcinoma, constitutive NF-kB activity in both CAFs and tumor cells is sustained by a positive mutual loop involving secreted IL-1β and the cognate receptor IL-1 receptor–associated kinase 4 (IRAK4), expressed on both cell types. Interestingly, CAF-conditioned medium is able to rescue PDAC cells from gemcitabine-induced apoptosis in vitro, and this protective effect is abrogated upon IRAK4 knockdown in CAFs. In PDAC mouse models, administration of either IL-1β-neutralizing antibodies or an IRAK4 inhibitor potentiates the effect of gemcitabine in suppressing tumor growth and fibrosis [86]. Further, IL-6 secreted by CAFs was reported to drive chemotherapy resistance in esophageal squamous cell carcinoma (ESCC). Briefly, IL-6 increases the expression of CXCR7 in ESCC cells via STAT3/NF-κB signaling, ultimately fostering their chemoresistant phenotype of ESCC cells upon treatment with cisplatin both in vitro and in subcutaneous xenografts. Consistently, CXCR7 expression is significantly higher in ESCC tissues from patients that had developed chemoresistance compared to chemosensitive ones [87]. Cisplatin treatment has also been shown to trigger AKT and ERK1/2 signaling pathways in ESCC cells in response to the release of Plasminogen activator inhibitor-1 (PAI-1) by CAFs. Activation of such pro-survival pathways exerts a protective effect against DNA damage, reactive oxygen species (ROS) accumulation and apoptosis. Both in vitro and in vivo analyses prove the efficacy of PAI-1 blockade, as shown by the synergistic effect of its inhibitor, Tiplaxtinin, combined with cisplatin. Finally, immunohistochemical staining of PAI-1 in samples from ESCC patients who receive cisplatin after surgery demonstrates a correlation between high PAI-1 expression in CAFs and a worse progression-free survival after chemotherapy [88].

Besides boosting pro-survival pathways in tumor cells, another important route to chemoresistance consists in supporting the CSC subpopulation, which is intrinsically resistant to cytotoxic drugs owing to its slow-cycling or quiescent state. One of the hallmarks of CSCs is indeed the ability to endure multiple insults, leading to therapy resistance [89, 90]. This “robustness” is partly due to cell-intrinsic mechanisms, but stromal cues are also crucial in inducing or maintaining stemness features as a mechanism of acquired resistance. Although colorectal cancer stem cells (CR-CSCs) display cell-autonomous resistance to chemotherapy, conditioned medium from chemo-treated human CAFs further enhances this phenotype through IL-17A -dependent activation of the NF-kB pathway and its downstream target ERK1/2 [82]. A different CAF-secreted mediator supporting chemotherapy resistance in CR-CSCs is TGFβ2, which induces non canonical SHH pathway in CSCs, thus sustaining stemness features through GLI2-driven transcription. HIF1α has been shown to cooperate with CAF paracrine signals to activate GLI2, which then promotes the resistance to 5-fluorouracil + oxaliplatin (FOX) therapeutic regimen. Furthermore, in patients’ data sets, sustained expression of TGFβ2/GLI2/HIF1α correlates with relapse after chemotherapy, further highlighting the therapeutic potential of TGFβ2 and GLI2 targeting [91]. In breast and lung cancer, a survival niche for CSCs is provided through IL-6 and IL-8 secretion by CD10+/GPR77+ fibroblasts, a functionally distinct subset enriched in biopsies of chemoresistant tumors prior to chemotherapy [11]. Furthermore, in breast cancer and PDAC, CAF secretion of ELR motif–positive (ELR+) chemokines following neoadjuvant chemotherapy has been shown to push CXCL2^+^ cancer cells toward a stem cell status with high invasive features [84].

As already pointed out, in the complex crosstalk between different cell types within a tumor, also the behavior of stromal cells can be shaped by the interaction with cancer cells. Release of the Hedgehog ligand by cancer cells can stimulate CAFs to produce a supportive niche via the secretion of FGF5 in triple negative breast cancer (TNBC). In TNBC mouse models, the use of Smoothened (Smo) inhibitors is able to revert this cascade of signals, reducing stemness features of tumor cells and increasing sensitivity to docetaxel, thus limiting the metastatic burden [92]. The mutual reprogramming of cancer and stromal cells is generated by an intricate circuitry of paracrine and autocrine signals that are the main determinants (together with genetic aberrations) of cancer onset, progression and clinical behavior. In breast cancer, the crosstalk with CAFs through PDGF-CC is a main determinant of the molecular subtype and blocking PDGF-CC is sufficient to revert basal-like resistant tumors into an ERα-positive subtype that responds to endocrine therapies [93]. Specifically, basal-like cancer cells express sustained levels of PDGF-CC, which stimulates CAFs to secrete stanniocalcin1 (STC1), HGF and insulin growth factor binding protein 3 (*IGFBP3*). In a feedback loop, the concerted action of these factors is able to suppress luminal-like features in cancer cells and sustain resistance to tamoxifen.

**Fig. 2:**
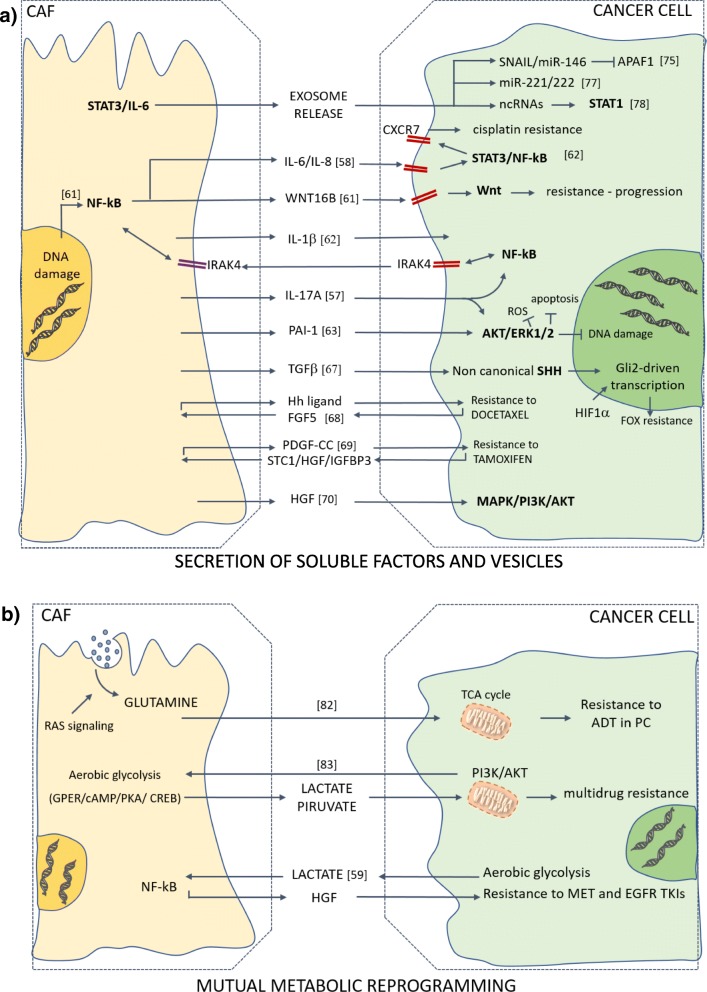
CAFs promote resistance to anti-cancer therapies through paracrine signals and mutual metabolic reprogramming. Upon exposure to a therapeutic insult, CAFs support an adaptive response in cancer cells that ultimately leads to therapy failure. **a**) Drug treatment triggers NF-kB and JAK/STAT signaling in CAFs. CAFs-released paracrine signals include exosome-mediated delivery of mRNAs and ncRNAs and a broad range of cytokines (mainly interleukins and growth factors). Activated pathways in cancer cells include pro-survival, anti-apoptotic and stemness programs. Signaling loops are depicted with rectangular-shaped arrows. **b**) As a mechanism of mutual adaptation to low levels of glutamine and glucose, CAFs provide metabolites that boost mitochondrial metabolism in cancer cells, hence fueling a resistant phenotype. Metabolites can also function as signaling molecules, as for the lactate secreted by cancer cells that induces NF-kB-mediated transcription in CAFs, which results in secretion of HGF that mediates TKIs resistance

TME-driven drug resistance is not restricted to conventional DNA-damaging chemotherapy, but rather concerns compounds that rely on different mechanisms of action, included oncogene-targeted drugs. The mechanisms underlying acquired resistance to targeted therapies have so far been explored through genomic profiling of tumor cells, which led to the identification of genetic alterations either in the target itself (“on-target” resistance) or in other downstream or parallel pathways (“off-target” resistance) that eventually compensated for the drug-inhibited oncogene. Hence, the contribution of the tumor-stroma interplay to non-cell-autonomous mechanisms of resistance to targeted agents has been underestimated. Recent evidence has shed light on the role of CAF-derived paracrine signals in conveying resistance to epidermal growth factor receptor (EGFR) targeted therapy. A co-culture screening has been employed to show that several stromal cell types secrete signals responsible for resistance to drugs, particularly to oncogene-targeted therapeutics [94]. HGF has been described as the main mediator of stroma-induced resistance to BRAF inhibitors in BRAF mutated melanoma, colorectal cancer (CRC) and glioblastoma, by activating MAPK and PI3K/Akt signaling in tumor cells via MET receptor [70]. Interestingly, a parallel MET signaling cascade triggered by CAFs-derived HGF was unveiled in KRAS^wt^ colorectal CSCs developing resistance to EGFR inhibition [95]. Although KRAS^wt^ CSCs isolated from xenografts are intrinsically sensitive to EGFR targeting, exposure to CAF-conditioned medium impairs the pro-apoptotic effect of cetuximab and gefitinib. Further, concomitant administration of cetuximab and MET inhibitor (JNJ-38877605) results in a more pronounced tumor regression compared to cetuximab monotherapy in vivo. Noteworthy, HGF expression in a public dataset of human KRAS^wt^ metastatic CRCs who progressed on cetuximab is significantly higher compared to responders. Overall, these findings identify a non-cell-autonomous mechanism of acquired resistance that contributes to relapse of KRAS^wt^ metastatic CRC patients under EGFR targeted therapy, thereby underscoring the inadequacy of the mutational status in predicting therapeutic outcome. In cholangiocarcinoma (CCA), a different mechanism of CAFs-induced resistance to EGFR tyrosine kinase inhibitors (TKIs) has been described. Briefly, CCA cells chronically treated with erlotinib exhibit an up-regulation of insulin receptor (IR)/insulin-like growth factor 1 receptor (IGF1R) signaling. Mechanistically, a positive feedback circuitry involving IR/IGF1R signaling and CAF-secreted IGF2 fuels both erlotinib resistance in CCA cells and activation of hepatic myofibroblasts. Accordingly, combined treatment with erlotinib and an IR/IGF1R inhibitor impairs growth of resistant tumor xenografts and reduces their stromal content [96] (Fig. 2a).

### Delivery of exosomal vesicles

Besides the secretion of soluble factors, the release of exosomal vesicles is crucial to vehicle paracrine signals that drive cancer cell aggressiveness and therapy resistance. Exosomes are membrane vesicles of 30–100 nm in diameter that contain proteins, DNA, mRNAs and miRNAs. Secreted exosomes are uptaken by neighboring cells via endocytosis and vesicle content is released into the cytoplasm of recipient cells. Exosome-delivered RNAs have been described as pivotal mediators of tumor progression and resistance and powerful biomarkers [97–99]. More recently, the role of exosome transfer in TME-orchestrated resistance has been highlighted. In PDAC, treatment with gemcitabine stimulates in resistant CAFs the secretion of exosomes that deliver SNAIL mRNA and its transcriptional target miR-146, thus conferring resistance to recipient cancer cells [100]. In ovarian cancer, miR-21 transfer from CAFs and Cancer Associated Adipocytes (CAAs) to cancer cells, stimulates cell motility and inhibits apoptosis thus enhancing chemoresistance, through its direct target apoptotic protease activating factor-1 (APAF1) [101]. Further, a mouse model of hormonal therapy resistance in luminal breast cancer has been exploited to elucidate the role of CAF-derived exosomes. Autocrine IL-6/STAT3 signaling fuels CAFs proliferation and stimulates the horizontal transfer of miR-221/222^high^ microvescicles to cancer cells. The uptake of miR-221/222 determines the induction of Notch-mediated CD133^high^ phenotype, which is responsible for resistance. IL-6 targeting abrogates this circuitry, hence blocking resistance to hormone therapy (HT). This mechanism is recapitulated also in CAFs derived from patients’ bone metastases [102]. Interestingly, in breast cancer the release of exosomes by stromal components determines the transfer of many non-coding RNAs (ncRNAs) and transposable elements to cancer cells, which activate STAT1-mediated antiviral response. Moreover, juxtacrine signaling of neighboring stromal cells induces the activation of the NOTCH3 pathway that converges into the STAT1 activation. These responses are able to select cancer cell subpopulations, enriching for tumor-initiating cells resistant to therapies [103].

In conclusion, exosomal transfer, together with other paracrine and juxtacrine signals, constitutes a major communication channel exploited by CAFs and other stromal components to sustain tumor progression and chemoresistance.

### Metabolic reprogramming of tumor cells

Tumor cells mainly rely on glutamine and glucose as energy sources and hijack CAF metabolism in order to meet their metabolic needs. Metabolic coupling between tumor cells and CAFs has been described as a mechanism of mutual adaptation to low nutrients availability that could be harnessed for novel therapeutic approaches [104–106]. Here, we will focus on the implications of such complementary metabolic reprogramming for the outcome of existing therapeutic strategies. Multidrug resistance can be triggered in cancer cells by the exchange of metabolites with surrounding CAFs that act as signal molecules inducing specific programs as differentiation or metabolic switches. For instance in prostate cancer, increased glutamine synthesis following macropinocytosis of extracellular fluid has been detected in primary CAFs and correlates with constitutive activation of Ras signaling [107]. In turn, CAF-secreted glutamine fuels prostate cancer mitochondrial metabolism and induces neuroendocrine differentiation, orchestrating an adaptive response to androgen signaling deprivation therapy (ADT). Consistently, greater blood glutamine levels have been detected in prostate cancer patients who progressed on ADT compared to responders. Notably, counteracting the uptake of stromal glutamine restores sensitivity to ADT in a castration-resistant xenograft model.

As a mechanism of adaptation to a glucose-deprived microenvironment, a metabolic switch towards aerobic glycolysis, known as Warburg effect, occurs in cancer cells. Interestingly, it has been reported that cancer cells can induce aerobic glycolysis in stromal cells, activating a loop that results in multidrug resistance [108]. Specifically, breast cancer cells with active PI3K/Akt signaling induce the Warburg effect in adjacent CAFs, via cytoplasmic translocation of the nuclear G-protein-coupled estrogen receptor (GPER) and the aberrant activation of a GPER/cAMP/PKA/CREB signaling axis. The extra pyruvate and lactate provided by glycolytic CAFs boost cancer cell metabolism and confer multidrug resistance. Accordingly, both chemotherapy- and tamoxifen-resistant tumor samples show a strong GPER cytoplasmic expression associated with an elevated metabolic activity in both local and metastatic sites, as measured by positron emission tomography/computed tomography (PET/CT). However, how cancer cells can instruct CAFs to trigger resistance-mediating pathways is poorly understood. The study from Apicella and colleagues shed light on a metabolism-based mechanism of adaptive resistance to MET and EGFR TKIs [83]. An in vivo model of adaptive resistance to MET TKIs was generated by long term administration of the maximum tolerated dose of a MET inhibitor in mice bearing a subcutaneous tumor xenograft of a non-small cell lung cancer (NSCLC) cell line, until resistance onset. Interestingly, tumor cells isolated from resistant xenografts are not intrinsically resistant in vitro but maintain the ability to reproduce resistant tumors upon re-injection, hinting at the involvement of cancer-derived signals activating the surrounding stroma. Indeed, resistant cells display a metabolic reprogramming towards aerobic glycolysis resulting in the production of high amounts of lactate. Lactate functions as the signaling molecule instructing CAFs to secrete HGF, the soluble cue responsible for the induction of MET TKI resistance in tumor cells, as previously reported [109]. Consistently, either pharmacologic or genetic targeting of lactate metabolism in tumor cells isolated from resistant xenografts completely prevents the onset of resistance to MET inhibition upon subcutaneous re-injection. Importantly, the role of the lactate-HGF axis in mediating adaptive resistance has been recapitulated for the EGFR TKI erlotinib, suggesting that the previous results can be applied to other oncogene-addicted lung cancer subtypes. Accordingly, an increased production of tumor lactate and stromal HGF were detected in advanced NSCLC patients upon the emergence of resistance to EGFR TKIs currently used in clinical practice (erlotinib and gefitinib), thus corroborating the clinical relevance of the reported findings.

Collectively, compelling experimental evidence has indicated coupled metabolic reprogramming of tumor cells and associated CAFs as a mechanism of mutual adaptation to therapeutic pressure, thus underscoring the need for targeting strategies aimed at sensitizing to conventional therapies (Fig. 2b).

## Targeting CAFs to hit cancer progression

CAFs are major players in driving onset and progression of solid tumors by affecting cancer cells’ plasticity, invasion and colonization ability, and therapeutic response. Their diverse tumor-supportive roles, combined with genetic stability and relative abundance among stromal cells, make these tumor cells’ henchmen an appealing therapeutic target. Here, we will briefly highlight the major advances and challenges in the development of CAF-directed anti-cancer therapies, although we recommend the recent review by Chen and Song for a more extensive dissertation on this topic [5]. Several anti-cancer strategies aiming at depleting the CAF population have been developed so far, ranging from metronomic chemotherapy to immune-based therapies. The traditional maximum-tolerated dose chemotherapy regimen has been reported to induce CAF secretion of chemokines that endow tumor cells with CSC traits, ultimately fostering chemoresistance. In contrast, metronomic chemotherapy, which consists in administering low doses of drug on a more frequent or continuous schedule, prevents CAF paracrine signaling and results in enhanced treatment response [84]. Moreover, DNA vaccines targeting FAP have succeeded in boosting CD8^+^ T cell-mediated killing of CAFs in pre-clinical studies. Remarkably, combining FAP vaccination with chemotherapy yielded up to 70% greater uptake of chemotherapeutic drugs in tumor xenografts [110]. More recently, co-administration of a novel FAP immunogen with tumor antigen-specific DNA vaccines synergistically enhanced antitumor immunity in mouse models of lung and prostate cancer [111]. As an alternative immune-based targeting strategy, adoptive transfer of FAP-specific chimeric antigen receptor (CAR) T cells proved to be effective in restraining tumor growth in pre-clinical models of lung, mesothelioma and pancreatic cancer [112–114]. However, the feasibility of the aforementioned approaches has been challenged by the finding that FAP^+^ cells reside in almost all tissues of the adult mouse and exert a pivotal function in preserving tissue homeostasis in the skeletal muscle and in the bone marrow [115].

Noteworthy, the identification of a tumor-suppressive role of CAFs has added a further layer of complexity [116, 117]. The recent identification of the cell surface markers (CD10 and GPR77) specifically defining the CAF subtype responsible for chemoresistance in breast and lung cancer represented a breakthrough in the field [11]. Selective targeting of such CAF subset with a GPR77-neutralizing antibody proved to be effective in enhancing tumor chemosensitivity in a patient-derived xenograft (PDX) model. Alternative promising therapeutic options include blockade of the pathways activated in CAFs that fuel the resistant phenotype in tumor cells. For instance, a Smoothened inhibitor hitting the activated Hedgehog signaling in CAFs successfully synergized with docetaxel chemotherapy in a phase I clinical trial enrolling TNBC patients [92]. Moreover, reprogramming activated CAFs into quiescent fibroblasts holds great promise. Vitamin D receptor (VDR) was identified as a druggable master regulator of the transcriptional program orchestrating the activation of pancreatic stellate cells [118]. Noteworthy, combined treatment with a VDR ligand and gemcitabine in a GEM model of pancreatic cancer resulted in dampened stromal inflammation and fibrosis, improved tumor uptake of gemcitabine and a 57% increase in survival compared to chemotherapy alone. Blunting of CAFs’ activation has been also achieved in bladder and pancreatic desmoplastic tumors upon treatment with nanoparticles loaded with a secretable TNF-related apoptosis-inducing ligand (sTRAIL). Secretion of sTRAIL by CAFs upon nanoparticles uptake has proved to be effective in counteracting tumor growth by exerting a dual function. In fact, by triggering apoptosis of adjacent tumor cells, it also impairs activation of residual fibroblasts owing to consequent lack of cancer cell-derived TGFβ in the tumor milieu [119].

## Conclusions

It is nowadays commonly accepted the notion that solid tumors are complex entities where transformed cells and stromal components coexist and influence each other in a kind of symbiotic relationship. Hitting transformed cells within their protective niche turns out much more complicated than expected, due to the unraveled role of ancillary cells. This scenario urges the need of reliable pre-clinical models able to mimic the network of interactions that are key determinant of cancer cells behavior and response to therapy. Cancer associated fibroblasts are one of the major components of tumor stroma and exert mainly a supportive role in the different steps of cancer lifespan, from the onset to the escape-dissemination phase and ultimately to the colonization of distant organs and resistance to therapies. Here, we have summarized the most recent and significant findings on the role of CAFs, with the intent to elucidate the mechanisms underlying their crosstalk with cancer cells and the clinical outcome of this mutual communication. CAFs are able to stimulate pro-survival and self-renewal programs in cancer cells by different mechanisms, mainly through the release of secreted paracrine factors (cytokines, exosomal vescicles, metabolites), but also by physical remodeling of the extracellular matrix, which ends up in a boosted motility of cancer cells that are therefore more prone to metastasize. Conversely, cancer cells actively shape CAF subpopulations to hijack their metabolism in order to sustain their survival and expansion. The close interaction between CAFs and transformed cells can strongly influence the clinical response to therapeutic regimens, as stromal signals foster an adaptive response of cancer cells to stress, like drug administration or oxygen/nutrients deprivation. In this scenario, targeting CAFs becomes an intriguing strategy that may synergize with standard anti-tumoral approaches to target more effectively cancer. Noteworthy, the identification of diverse subtypes of CAFs and the lack of unique markers that identify these subpopulations added a further degree of complexity. Therefore, the translation of the reported pre-clinical efforts into clinical practice claims a better molecular characterization of CAFs’ heterogeneity, in order to develop tailored therapeutic approaches able to selectively eradicate a specific CAF subset. Moreover, despite the large body of evidence focusing on the understanding of CAFs biology, it is important to notice that most of the studies are based on in vitro assays, which may give rise to possible artifacts since the culture conditions may alter the paracrine activity of CAFs [120]. For this reason, the use of multiple cell surface markers would be preferable for the isolation of CAFs from patients’ samples, rather than selection based on their survival advantage in culture medium. A possible further source of artifacts and misleading results is the limited lifespan of primary CAF cultures, before replicative senescence occurs, that may strongly impair data reproducibility. Moreover, a major challenge for the in vivo study of CAFs is represented by the lack of an established GEM model that allows for in vivo CAFs tracking and a reliable imaging tool to discriminate CAFs’ dynamics during cancer progression. Furthermore, more efforts should be addressed to unravel the crosstalk between CAFs and other important stromal players, such as immune cells and endothelial cells, to finally draw a complete picture of the TME contribution to tumor biology.

## Abbreviations

ADT: Androgen signaling deprivation therapy
APAF1: Apoptotic protease activating factor 1
BMDCs: Bone marrow-derived cells
CAAs: Cancer-associated adipocytes
CAFs: Cancer-associated fibroblasts
CAR: Chimeric antigen receptor
CCA: Cholangiocarcinoma
CM: Conditioned medium
CRC: Colorectal cancer
CSCs: Cancer stem cells
ECM: Extracellular matrix
EGFR: Epidermal growth factor receptor
EMT: Epithelial-to-mesenchymal transition
EndMT: Endothelial-to-mesenchymal transition
ESCC: Esophageal squamous cell carcinoma
FAP: Fibroblast activation protein
FGF: Fibroblast growth factor
FN1: Fibronectin 1
FOX: 5-fluorouracil-oxaliplatin
FSP1: Fibroblast specific protein 1
GEMM: Genetically engineered mouse model
GPER: G-protein-coupled estrogen receptor
HGF: Hepatocyte growth factor
HOTAIR: HOX transcript antisense RNA
HT: Hormone therapy
IGF1R: Insulin-like growth factor 1 receptor
IGFBP3: Insulin growth factor binding 3
IL-6: Interleukin-6
IR: Insulin receptor
IRAK4: IL-1 receptor-associated kinase 4
lncRNA: Long non-coding RNA
MICs: Metastasis initiating cells
MMPs: Matrix metalloproteinases
MRD: Minimal residual disease
NAFs: Normal activated fibroblast
ncRNA: Non-coding RNA
NFs: Normal fibroblasts
NSCLC: Non-small cell lung cancer
OPN: Osteopontin
OSCC: Oral squamous cell carcinoma
PAI-1: Plasminogen activator inhibitor 1
PDAC: Pancreatic ductal adenocarcinoma
PDGFR: Platelet-derived growth factor receptor
PDX: Patient-derived xenograft
PET/CT: Positron emission tomography/computed tomography
PIP_3_: Phosphatidylinositol (3,4,5)-trisphosphate
POSTN: Periostin
PTK7: Protein tyrosine-kinase 7
ROS: Reactive oxygen species
RTK: Receptor tyrosine- kinase
SDF1: Stromal derived factor
SHH: Sonic the hedgehog
Smo: Smoothened
SPARC: Secreted protein acidic and rich in cysteine
STC1: Stanniocalcin 1
sTRAIL: Secretable TNF-related apoptosis-inducing ligand
TGFβ: Transforming growth factor-β
THSB2: Thrombospondin-2
TKI: Tyrosine kinase inhibitors
TME: Tumor microenvironment
TNBC: Triple negative breast cancer
TSP: Thrombospondin
VDR: Vitamin D receptor
αSMA: Alpha smooth muscle actin
